# Supplementary material for: Low-intensity pulsed ultrasound promotes mesenchymal stem cell transplantation-based articular cartilage regeneration via inhibiting the TNF signaling pathway
Source: Stem Cell Res Ther. 2023 Apr 17;14:93. doi: 10.1186/s13287-023-03296-6 (PMC10111837; doi:10.1186/s13287-023-03296-6)
Supplement: Supplementary file 1 — Additional file 1 Table S1: Lists of hUC-MSCs qPCR primers. Table S2: Lists of C28/I2 qPCR primers [file 13287_2023_3296_MOESM1_ESM.pdf]

Additional File 1 for:

Low-intensity pulsed ultrasound promotes mesenchymal stem cell transplantation-based articular cartilage regeneration via inhibiting the TNF signaling pathway

Yiming Chen[1†], Huiyi Yang[2†], Zhaojie Wang[2,4†], Rongrong Zhu[2,4], Liming Cheng[2*], Qian Cheng[1,2,3*]

[1] Institute of Acoustics, School of Physics Science and Engineering, Tongji University, Shanghai 200092, China.

[2] Key Laboratory of Spine and Spinal Cord Injury Repair and Regeneration of Ministry of Education, Department of Orthopedics, Tongji Hospital affiliated to Tongji University School of Medicine, Tongji University, Shanghai 200065, China.

[3] Frontiers Science Center for Intelligent Autonomous Systems, Shanghai 201210, China.

[4] School of Life Science and Technology, Tongji University, Shanghai 200065, China.

[†]These authors have contributed equally to this work and share first authorship

* Corresponding author: Qian Cheng, Liming Cheng

E-mail address: q.cheng@tongji.edu.cn (Q. Cheng), limingcheng@tongji.edu.cn (L. Cheng)

Table S1. Lists of hUC-MSCs qPCR primers.

Gene	Sense Strand (5'-3')	Antisense Strand (5'-3')
GAPDH	CTCCTCACAGTTGCCATGTA	GTTGAGCACAGGGTACTTTATTG
SOX-9	ACCTTTGGGCTGCCTTATATT	TCCCTCACTCCAAGAGAAGAT
COL-II	CAAACCCAAAGGACCCAAGTA	TGTGAGAGGGTGGGATGAA
COL-X	ACCCAAGGACTGGAATCTTTAC	GCCATTCTTATACAGGCCTAC
ACAN	AGTCCTCAAGCCTCCTGTACTCA	CGGGAAGTGGCGGTAACA
COMP	CGAGTCCGCTGTATCAACACC	TCCGTGCAAACCTGCTTGT
TNF-α	TCAGAGGGCCTGTACCTCAT	GGAGGTTGACCTTGGTCTGG
IL-1β	TGAGCTCGCCAGTGAAATGA	CATGGCCACAACAACTGACG
CXCL8	CAGTTTTGCCAAGGAGTGCT	AGTTTTCCTTGGGGTCCAGA

GAPDH, glyceraldehyde 3-phosphate dehydrogenase; *SOX-9*, sex-determining region Y-box 9; *COL-II*, type II collagen; *COL-X*, type X collagen; *ACAN*, aggrecan; *COMP*, cartilage oligomeric matrix protein; *TNF-α*, tumor necrosis factor-α; *IL-1β*, interleukin-1β; *CXCL8*, C-X-C motif chemokine ligand 8.

Table S2. Lists of C28/I2 qPCR primers.

Gene	Sense Strand (5'-3')	Antisense Strand (5'-3')
GAPDH	GGTCGGTGTGAACGGATTTGG	GCCGTGGGTAGAGTCATACTGGAAC
TNF-α	ATGCTGGGACAGTGACCTGG	ATTGTCATACCAGGAAATGAGCTT
IL-1β	CCAAAAGATGAAGGGCTGCT	CCTTGATGGTGGTGCATGAG
IL-6	TTCCATCCAGTTGCCTTCTTG	GAAGGCCGTGGTTGTCACC

GAPDH, glyceraldehyde 3-phosphate dehydrogenase; *TNF-α*, tumor necrosis factor-α; *IL-1β*, interleukin-1β; *IL-6*, interleukin 6.
