# Supplementary material for: Low-intensity pulsed ultrasound promotes mesenchymal stem cell transplantation-based articular cartilage regeneration via inhibiting the TNF signaling pathway
Source: Stem Cell Res Ther. 2023 Apr 17;14:93. doi: 10.1186/s13287-023-03296-6 (PMC10111837; doi:10.1186/s13287-023-03296-6)
Supplement: Supplementary file 2 — Additional file 2 Figure S1: Representative Alcian blue staining images for the chondrocyte spheres in suspension culture for LIPUS stimulation and adhered for staining. Figure S2: Western blot data of chondrogenic proteins (COL-II, ACAN, and SOX-9) and actin after different parameters of LIPUS stimulation [file 13287_2023_3296_MOESM2_ESM.pdf]

Additional File 2 for:

Low-intensity pulsed ultrasound promotes mesenchymal stem cell transplantation-based articular cartilage regeneration via inhibiting the TNF signaling pathway

Yiming Chen[1†], Huiyi Yang[2†], Zhaojie Wang[2,4†], Rongrong Zhu[2,4], Liming Cheng[2*], Qian Cheng[1,2,3*]

[1] Institute of Acoustics, School of Physics Science and Engineering, Tongji University, Shanghai 200092, China.

[2] Key Laboratory of Spine and Spinal Cord Injury Repair and Regeneration of Ministry of Education, Department of Orthopedics, Tongji Hospital affiliated to Tongji University School of Medicine, Tongji University, Shanghai 200065, China.

[3] Frontiers Science Center for Intelligent Autonomous Systems, Shanghai 201210, China.

[4] School of Life Science and Technology, Tongji University, Shanghai 200065, China.

[†]These authors have contributed equally to this work and share first authorship

* Corresponding author: Qian Cheng, Liming Cheng

E-mail address: q.cheng@tongji.edu.cn (Q. Cheng), limingcheng@tongji.edu.cn (L. Cheng)

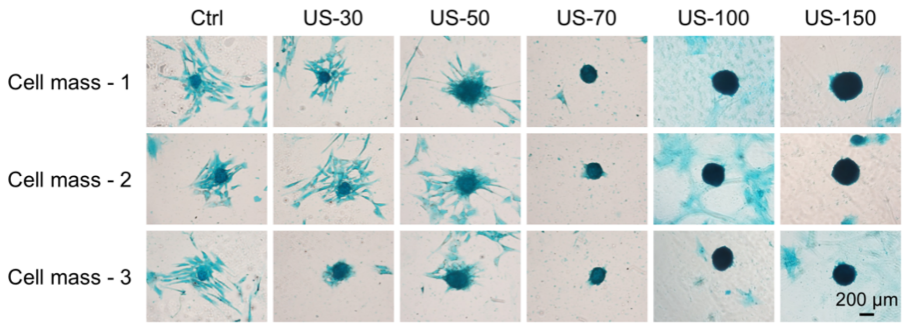

Figure S1. Representative alcian blue staining images for the chondrocyte spheres in suspension culture for LIPUS stimulation and adhered for staining; scale bar: 200 μm.

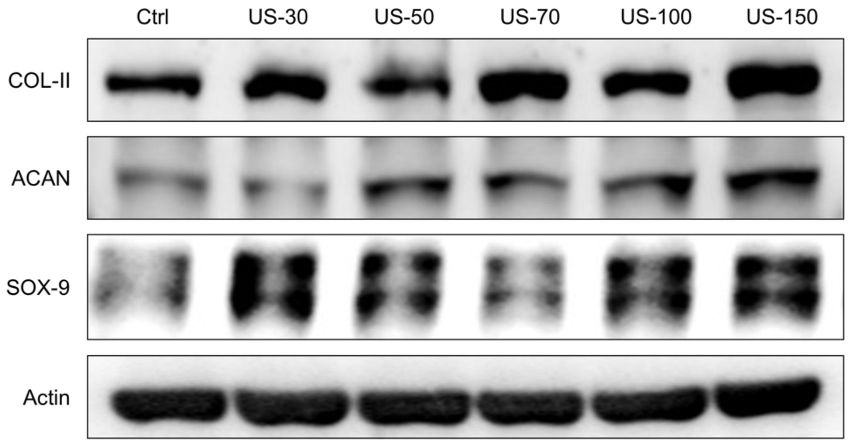

Figure S2. Western blot data of chondrogenic proteins (COL-II, ACAN, and SOX-9) and Actin after different parameters of LIPUS stimulation. Full-length blots are presented in Additional File 3.
