# Supplementary material for: Low-intensity pulsed ultrasound promotes mesenchymal stem cell transplantation-based articular cartilage regeneration via inhibiting the TNF signaling pathway
Source: Stem Cell Res Ther. 2023 Apr 17;14:93. doi: 10.1186/s13287-023-03296-6 (PMC10111837; doi:10.1186/s13287-023-03296-6)
Supplement: Supplementary file 3 — Additional file 3 Full-length blots of the data shown in Figure S2. [file 13287_2023_3296_MOESM3_ESM.pdf]

Additional File 3 for:

Low-intensity pulsed ultrasound promotes mesenchymal stem cell transplantation-based articular cartilage regeneration via inhibiting the TNF signaling pathway

Yiming Chen[1†], Huiyi Yang[2†], Zhaojie Wang[2,4†], Rongrong Zhu[2,4], Liming Cheng[2*], Qian Cheng[1,2,3*]

[1] Institute of Acoustics, School of Physics Science and Engineering, Tongji University, Shanghai 200092, China.

[2] Key Laboratory of Spine and Spinal Cord Injury Repair and Regeneration of Ministry of Education, Department of Orthopedics, Tongji Hospital affiliated to Tongji University School of Medicine, Tongji University, Shanghai 200065, China.

[3] Frontiers Science Center for Intelligent Autonomous Systems, Shanghai 201210, China.

[4] School of Life Science and Technology, Tongji University, Shanghai 200065, China.

[†]These authors have contributed equally to this work and share first authorship

* Corresponding author: Qian Cheng, Liming Cheng

E-mail address: q.cheng@tongji.edu.cn (Q. Cheng), limingcheng@tongji.edu.cn (L. Cheng)

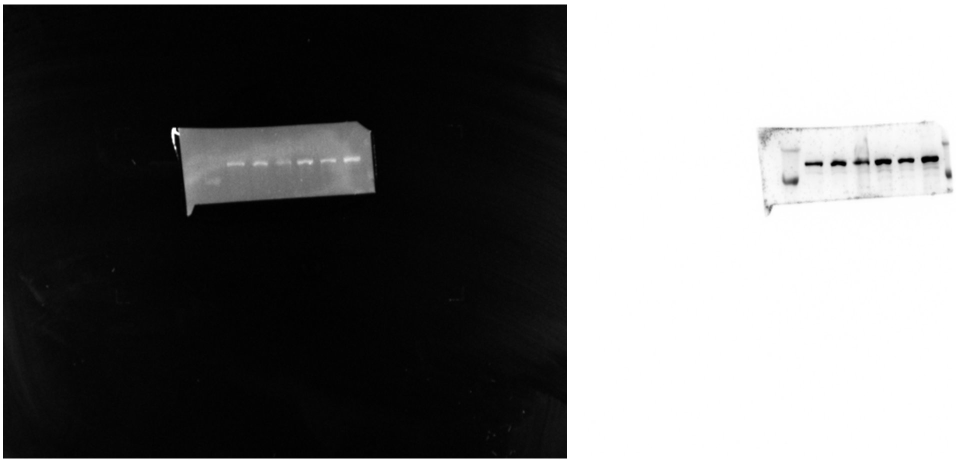

COL-II. Original blot image of COL-II after different parameters of LIPUS stimulation. Combined figures are presented in Figure S2.

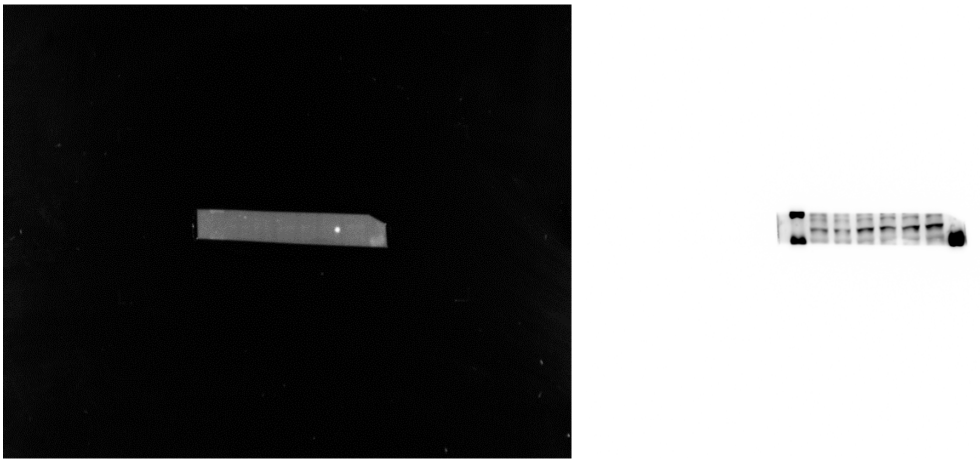

ACAN. Original blot image of ACAN after different parameters of LIPUS stimulation. Combined figures are presented in Figure S2.

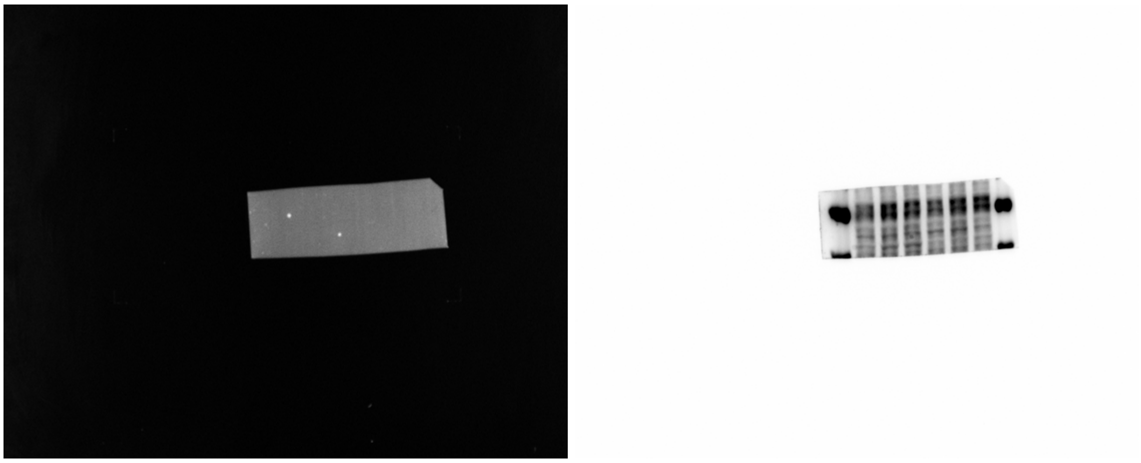

SOX-9. Original blot image of SOX-9 after different parameters of LIPUS stimulation. Combined figures are presented in Figure S2.

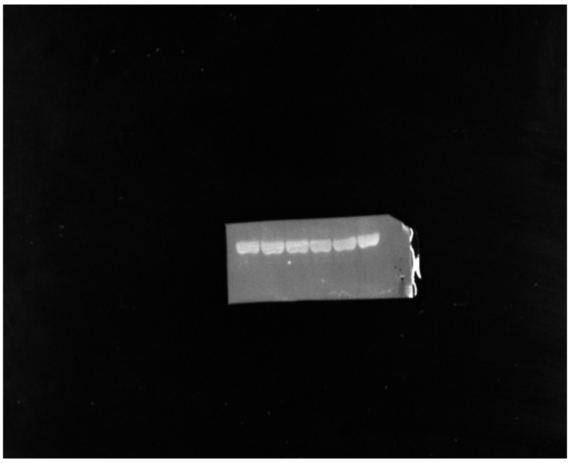

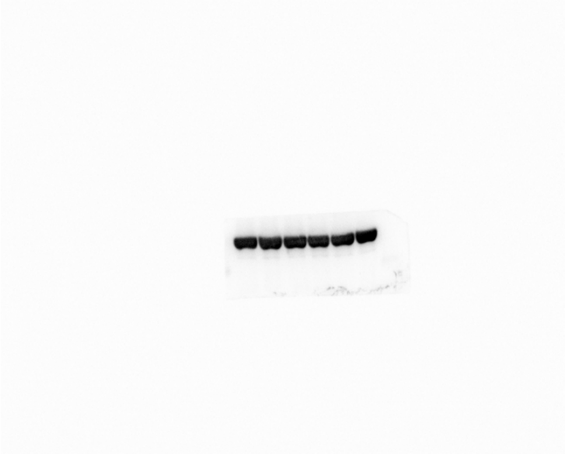

Actin. Original blot image of Actin after different parameters of LIPUS stimulation. Combined figures are presented in Figure S2.
